# Isolated Congenital Camptodactyly and Temporomandibular Joint Articular Disc Displacement

**DOI:** 10.7759/cureus.31658

**Published:** 2022-11-18

**Authors:** Christina A Aponte, Adrienne Hoffman, Selvana Moussa, Nicholas Norvell, Zaid H Khoury

**Affiliations:** 1 Orthodontics, BronxCare Health Systems, New York, USA; 2 Oral Diagnostic Sciences & Research, Meharry Medical College School of Dentistry, Nashville, USA; 3 Prosthodontics, Meharry Medical College School of Dentistry, Nashville, USA

**Keywords:** tinnitus, temporomandibular joint disorder, rehabilitation, syndrome, orthognathic surgery, camptodactyly

## Abstract

The development of craniofacial structures is complex and involves multiple cellular and molecular interactions. We report a case of congenital camptodactyly in a female who subsequently developed chronic tinnitus and temporomandibular joint dysfunction. This report describes the clinicoradiographic features and surgical management of the facial skeletal manifestations, along with postoperative rehabilitation. Furthermore, a concise review of similar literature raises the question of whether this complex of manifestations represents a new entity or a minimal manifestation of a previously characterized syndrome. As such, a possible developmental association between camptodactyly and temporomandibular joint dysfunction is suggested.

## Introduction

Temporomandibular joint dysfunction (TMD) is a non-diagnostic general term that encompasses a set of conditions that alter the functional anatomy or physiology of neuro-musculo-skeletal components of the masticatory system [1]. The etiopathogenesis of these disorders can be complex and multifactorial, involving biological and psychological interactions [2]. Disorders affecting the articulating disc of the temporomandibular joints (TMJs) are categorized as disc displacement “with reduction,” “with reduction with intermittent locking,” “without reduction with a limited opening,” and “without reduction without a limited opening” [3].

Camptodactyly, a congenital anomaly of the hand affecting less than 1% of the population, is characterized by flexion of the proximal interphalangeal (PIP) joint resulting in finger deformity, most commonly in the fifth digit [4]. Trismus and TMDs as components of rare familial and nonfamilial camptodactyly syndromes have been recognized, suggesting a mutual underlying factor altering the developmental biology of the masticatory apparatus in addition to the PIP joint of the fingers [5-8].

This report highlights the case of a 25-year-old female with congenital bilateral camptodactyly who had a history of bilateral TMJs disc displacement. Surgical management and rehabilitation of the TMJs, along with a discussion focused on related literature, is presented in this concise report, raising the question of possible developmental associations between camptodactyly and TMDs.

## Case presentation

A 25-year-old Hispanic female patient presented to the clinic with a chief concern of bilateral tinnitus (more severe on the left side), mild jaw pain, mouth breathing, and bruxism. Upon intraoral examination, bilateral craze lines were noted on the maxillary canines with normal mouth opening (51 mm) with a deviation of the mandible to the left side upon opening. No other teeth or soft tissue abnormalities were detected; however, the patient mentioned that the alignment of her dentition has changed, which may be suggestive of an orthodontic treatment relapse. Extraoral examination revealed bilateral camptodactyly of the fifth digits (little fingers), which was more pronounced on the left hand. With further interviewing, the patient reported curved phalanges of the foot. The patient denied any familial inheritance pattern and was otherwise in good health.

**Figure 1:**
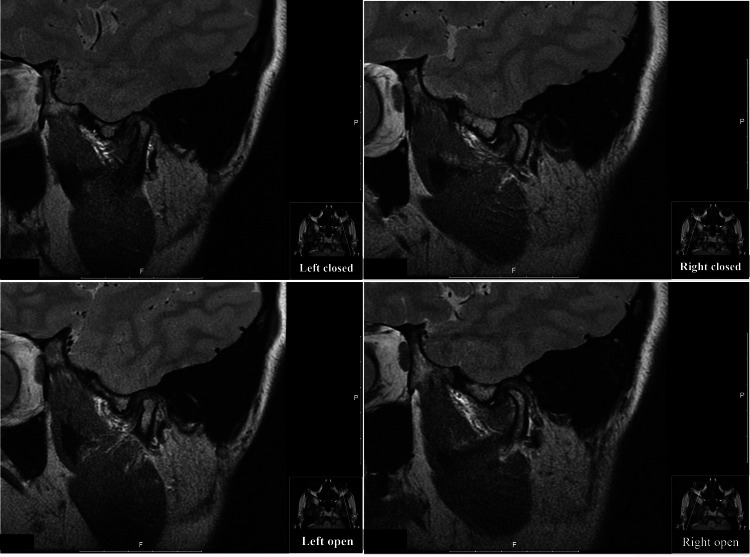
Magnetic resonance imaging of the left and right TMJ/temporal fossa regions. Sagittal closed mouth views (upper panels); sagittal open mouth views (lower panels). TMJ: temporomandibular joint.

**Figure 2:**
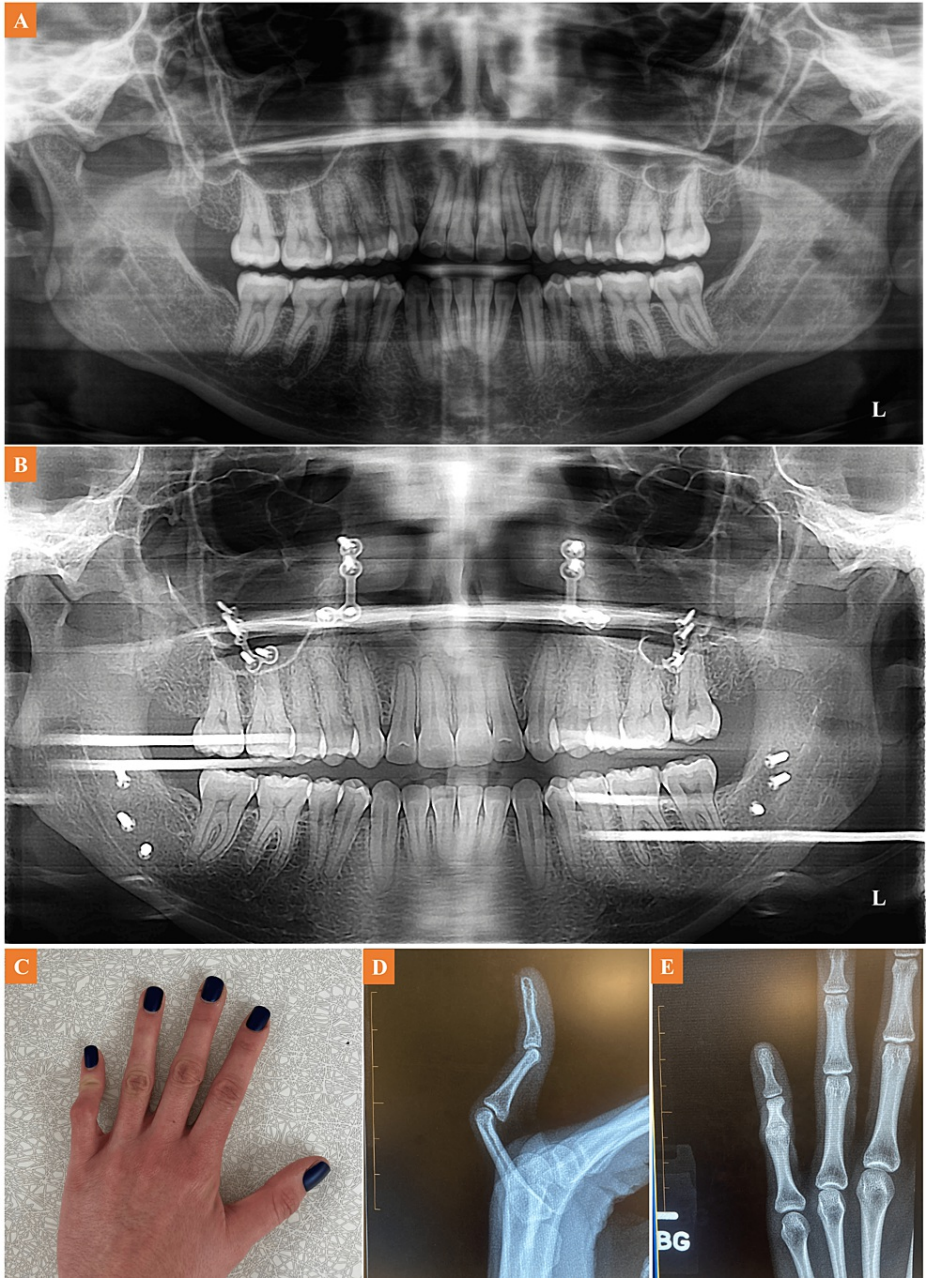
(A) Preoperative panoramic radiograph of the patient at 21-year-old; (B) post-operative panoramic radiograph of the patient at 22-year-old following LeFort 1 osteotomy and bilateral sagittal split osteotomy; (C) clinical picture of the left hand; (D) oblique radiographic view of the left hand; and (E) left hand posterior-anterior oblique radiographic view.

The patient's past dentoalveolar history was significant for the following reasons: the patient received orthodontic treatment between the ages of 10 and 12 to correct teeth malalignment and malocclusion. At 20 years old, the patient presented to an orthodontist's office with a persistent anterior open bite, malocclusion, and jaw pain. Imaging studies determined that the patient has a deviated septum with hypertrophic nasal conchae and the presence of a bony spur. She was then referred to an oral and maxillofacial surgeon for further evaluation. At age 21, it was determined by orthognathic specialists that the patient suffers from a stage 4B displacement of the condylar disc of the left and right TMJs. The magnetic resonance imaging (MRI) report indicated medial pole disc displacement without reduction and condylar surface irregularity of both joints (Figure 1). Additionally, the right TMJ was affected with condylar hypoplasia and effusion, while examination of the left TMJ revealed the presence of an osteophyte. The patient underwent orthognathic surgery consisting of a LeFort 1 osteotomy and bilateral sagittal split osteotomy (BSSO) to correct her TMJs disc displacements, anterior open bite, and class 2, division 1 malocclusion. At three weeks post-operatively, it was determined that the patient presented with a unilateral crossbite on the left side. The patient underwent another BSSO (Figure 2). The patient was under the care of a physical therapist and a chiropractor post-operatively to manage hypertrophy and tension of the masseter and trapezius muscles. Although it was offered as an option, the patient did not wish to proceed with botulinum toxin injections to promote muscle relaxation. No immediate complications were reported by the patient post-operatively, and the patient returned to normal function within two months.

Two years post-operatively, the patient began to complain of orthodontic movement in her anterior maxilla and tinnitus, along with a labial flare of the maxilla. At age 25, an otorhinolaryngologist corrected the deviated nasal septum and removed a bone spur present in the left septal region. Post-operatively, the patient was under the care of an acupuncturist. A prosthodontist has also fabricated a pivot appliance to mediate articular disc healing.

## Discussion

The patient’s TMD, in our case, did not result in limited mouth opening (trismus) per her previous records. Furthermore, our patient denied any known family history of similar manifestations. A review of related literature on patients presenting with both camptodactyly and TMD over the past four decades revealed that these manifestations occurred in the context of the auriculo-condylar syndrome (ACS) and trismus-pseudocamptodactyly syndrome (TPS) [5-7,9-14]. Table 1 summarizes the data and surgical management of the reviewed cases.

**Table 1:** A summary of reported cases demonstrating correlative incidences of camptodactyly and TMD. ACS: auriculo-condylar syndrome, TPS: trismus-pseudocamptodactyly syndrome, TMD: temporomandibular joint dysfunction.

Author-year	Cases	Age at initial presentation	Sex	Family history	Association	Surgical management of TMD
Vasquez-Colon et al., 2021 [14]	1	Six years	Male	Mother had similar manifestations	TPS	Bilateral coronoidectomies and release of masseter trismus
Kusano et al., 2018 [5]	1	Ten years	Male	Non-remarkable	TPS	Hypoplastic mandibular condyles requiring correction
Marianetti et al., 2014 [6]	1	Three years	Female	-	TPS	Bilateral coronoidectomies and subsequent surgeries for recurrent hyperplasia
Sreenivasan et al, 2013 [7]	1	Eight years	Male	Consanguineous marriage	TPS	Patient was advised to undergo surgery to remove masseter contracture and correct left condylar alterations
Papagrigorakis et al., 2012 [12]	1	Eleven years	Female	History of the syndrome among sister, father, and paternal family members (three generations)	ACS	Bilateral vertical mandibular ramus lengthening using distraction osteogenesis
Carlos et al., 2005 [9]	1	Six years	Male	Non-remarkable, unknown paternal family history	TPS	Bilateral coronoid amputation
Karras et al., 1995 [10]	1	Fourteen years	Female	-	TPS	Coronoidectomies and release of fibrosis to correct for severe fibrosis around hypertrophic coronoid processes
Markus, 1986 [11]	1	Twenty-three years	Male	Patient was adopted; unknown	TPS	Bilateral coronoidectomies
Tsukahara et al., 1985 [13]	1	Eight years	Male	Five family members through three generations (one affected from the first generation, two in the second and third, respectively)	TPS	-
	Four family members	-	-			

TPS (Hecht-Beals syndrome or Dutch-Kentucky syndrome) is caused by a mutation in the myosin heavy chain 8 (MYH8) gene affecting early skeletal development. All reviewed cases documented camptodactyly and trismus with or without coronoid hyperplasia [5,14]. In contrast, we identified only one report of a patient (with significant family history) affected with ACS who had concurrent camptodactyly and TMD. ACS results from first and second pharyngeal arches abnormalities during embryological development [12]. Collectively, this case represents isolated congenital camptodactyly in a patient that later developed TMD due to articular disc displacement with the absence of trismus. Whether this case represents a minimal manifestation of TPS or ACS requires further evaluation.

The developmental events that govern the formation of the TMJ articular disc and the PIP joint overlap in utero [15-18] (Table 2).

**Table 2:** Summary of the developmental events related to the PIP joints and TMJ articular disc formation in utero. PIP: proximal interphalangeal, TMJ: temporomandibular joint. Source [15-18].

Structure	Timeline
PIP joints	
Hand and footplates are formed. Hyaline cartilage framework and cartilaginous condensation forming joints commence. Thumb slot formation.	Six weeks
Muscles differentiate and insert into developing bones through ligaments followed by maturation.	Six to nine weeks
Rotation of the upper and lower limbs laterally and medially, respectively. Spatial plane of all fingers is established with the prominence of interdigital and digital ridges.	Seven to ten weeks
Regression of interdigital and digital ridges and creases appear. The interphalangeal flexion crease is the least to form. Endochondral ossification commences (12 weeks) and progresses toward the end of the cartilaginous framework.	Eleven to thirteen weeks
TMJs	
Rudimentary mandibular condyle formation.	Seven weeks
Mandibular ramus intramembranous ossification commences reaching the condylar area. Rudimentary articular disc formation.	Eight weeks
Mandibular condyle cartilage formation.	Nine weeks
Inferior joint cavity formed.	Ten weeks
Superior joint cavity formed.	Eleven weeks
Fibrous projections from the anterior articular disc are evident, connecting the disc to the temporalis and the masseter muscles.	Thirteen weeks

We hypothesize that due to the distal location of the TMJ complex and the PIP joint of the fifth digit from the axial skeleton, there may be a temporal relationship between causation and the resulting developmental anomalies. Our patient did not undergo genetic testing, and the possibility of a somatic mutation causing these manifestations should be considered. Presently, there is limited literature discussing the non-genetic association between camptodactyly and temporomandibular joint dysfunction (TMD), raising the question of whether individuals with congenital camptodactyly are at a higher risk of TMDs attributed to a developmental event.

A multidisciplinary team was involved in the patient’s rehabilitation post-operatively at ages 21 and 25. Our patient sought treatment through an acupuncturist, a prosthodontist, a physical therapist, and a chiropractor. She noted a release in muscle tension and a reduction in tinnitus through the care of her acupuncturist, with weekly visits. The prosthodontist offered her a pivot appliance that created a posterior separation of the dentition to allow for the articular disc to heal. The patient reported wearing the splint for about 22 hours per day for the first two months. Due to symptomatic relief, the patient used it only at night for another three to five months. This separation presumably caused a reduction in TMJ pain sensation and tinnitus severity. Weekly visits to the chiropractor offered no relief of symptoms of camptodactyly or in the TMJ region. However, the patient noted that chiropractic visits reduced overall upper back pain caused by the forward head posture developed from habitual mouth opening. The physical therapist offered the most benefit, as stated by the patient. The physical therapist began with mandibular stabilization and cervical retraction “chin tucks,” to improve the range of motion. However, while completing the “tongue up” exercise, where the patient is asked to make a clucking sound, it was determined that the patient’s jaw mobility is within normal limits and supported by minimal musculature. As such, the patient was asked to increase her side-to-side jaw exercises to stabilize the mandibular range of motion. The patient also received dry needling by her physical therapist, aimed at relaxing the trapezius and masseter muscles. A prosthodontist evaluated the patient for non-surgical temporomandibular joint dysfunction treatment options as adjunctive therapy to her previous treatments. While load-testing the temporomandibular joints with an anterior deprogrammer, the patient was positive for lingering pain in the joint space. Based on these symptoms, an oral appliance, the pivot appliance, was fabricated. This appliance provides intentional and therapeutic transfer of the mandibular rotational fulcrum from the TMJ to the appliance to unload or decompress the retrodiscal tissue until healing occurs [19,20]. To our knowledge, there is only one other case report in the context of camptodactyly discussing orthognathic surgery relapse, as outlined by Marianetti et al. [6]. In our case, however, no coronoid hyperplasia was detected.

## Conclusions

There are few cases published in the literature on the management of patients with congenital camptodactyly who subsequently developed TMD. In our case, a multidisciplinary team managed the patient over several years, which was accompanied by a significant psychological, functional, and financial burden. Well-controlled studies on an adequate cohort of patients with similar manifestations to our case are mandated to answer the following questions: Are patients with congenital camptodactyly more prone to the development of TMD? Is the anomaly responsible for the TMD symptomology of developmental origin? Answering these questions aid in investigating whether a temporal relationship of developmental origin contributes to these manifestations and whether such an event correlates with the cause of the TMD (anomalous coronoid process, articular disc, or mandibular condyle). This is significant for possible TMD early detection and proper referral in patients presenting with congenital camptodactyly.
